# Mr. Alcock

**Published:** 1833-10-01

**Authors:** 


### Mr. Thomas Alcock.

We regret to add to the above obituary, the decease of Mr. Thomas Alcock, of New Burlington-street, on the 21st of August, aged, we believe about 56—or 58. He is well known by several Essays, but especially by his paper on Medical Education, which excited some sensation, and of which we gave an account in this Journal. He was a gentleman of considerable talent, but of rather an eccentric disposition; and of very irritable temper, more especially of late years—probably dependent on the state of his health.

Mr. Alcock died a batchelor, and his nephew, who studied under him, has distinguished himself very much at Oporto, in the service of Don Pedro.

